# How Can We Increase Pro-environmental Behavior During COVID-19 Pandemic? Focusing on the Altruistic (vs. Egoistic) Concerns

**DOI:** 10.3389/fpsyg.2022.870630

**Published:** 2022-05-03

**Authors:** Yaeri Kim, Seojin Stacey Lee

**Affiliations:** ^1^Department of Data Science, Seoul Women’s University, Seoul, South Korea; ^2^Center for Happiness Studies, Seoul National University, Seoul, South Korea

**Keywords:** concern for ESG, egoistic COVID-19 concerns, altruistic COVID-19 concerns, pro-environmental behavior, environmental prompts, moderated mediation effect

## Abstract

Would the life-threatening pandemic impact pro-environmental behavior? This study demonstrates the effects of coronavirus disease 2019 (COVID-19) on pro-environmental product consumption. Two experimental studies manipulated individuals’ COVID-19 concerns and the presence/absence of pro-environmental prompts. In study 1, we found that consumers indicated lower purchase intention for a product with the environmental prompts when recalling COVID-19 concerns compared to normal situations. In study 2, we disentangled egoistic COVID-19 concerns (e.g., concerns about disadvantages to individuals’ work and finances) from altruistic COVID-19 concerns (e.g., concerns about damage to the country’s economy) and investigated the effects of both these concerns on pro-environmental product consumption. The results of study 2 revealed that consumers reported an increased purchase intention toward the e-prompt products, which manipulated altruistic COVID-19 concerns. However, the presence/absence of e-prompt products did not affect consumers’ purchase intentions when recalling egoistic COVID-19 concerns. Concerns regarding environmental, social, and corporate governance (ESG) issues mediated the interaction effect between the type of COVID-19 concerns and the presence/absence of e-prompts for the products.

## Introduction

Pro-Environmental behavior is fundamental for individuals and societies to maintain sustainable lives. Therefore, it is important to understand how pro-environmental behavior develops during a catastrophe and determine the factors that advance the deeds. The ongoing coronavirus disease 2019 (COVID-19) pandemic is the world’s most significant global disaster affecting individuals and nations worldwide (Word Health Organization, 2020). Since altruistic behavior benefits extend not only to society as a whole, but also to individuals who offer help as a form of relieving individuals’ stress (Raposa et al., 2016) and mitigating physical pain (Wang et al., 2020); it is meaningful to broaden our understanding of individuals’ pro-environmental behavior during this ongoing worldwide pandemic.

Extant literature supports the idea that crises such as COVID-19 can either elevate or lower prosocial behavior. For example, the empirical literature has demonstrated that behaviors directed toward others increase in difficult times, such as natural disasters (Rodriguez et al., 2006) and war (Bauer et al., 2016). In addition, when people are confronted with common challenges, cooperation increases, which is called the “common enemy effect” (Ostrom et al., 1999). On the other hand, other studies have suggested that crises prompt selfish and antisocial behavior because of a lack of resources and an increased competition (Dietz, 2003; Hsiang et al., 2013). Regardless of the effect, both the accounts show a shift in prosocial conduct during a crisis, either an increase or a decrease.

Despite the importance of prosocial behavior and its relationship with social crises such as COVID-19 pandemic, only a few studies have directly explored the potential relationship between the crisis and individual behavior on environmental responsibility (Tchetchik et al., 2021). For example, Urban and Braun Kohlová (2020) insisted that there is no statistically significant association between COVID-19 crisis and pro-environmental attitudes. More specifically, Lucarelli et al. (2020) suggested that individuals who are more aware of the relationship between COVID-19 and climate change have shown more instances of pro-environmental behavior. To provide a better understanding of the link between COVID-19 pandemic and pro-environmental behavior, we conducted two empirical studies that manipulated the individual COVID-19 concerns (Rojas-Méndez, 2021) and the presence/absence of pro-environmental prompts (Moussaoui et al., 2020) and examined the effects of both the factors on consumer behavior. We expected that individuals who were concerned about public issues evoked by the pandemic would be aware of corporate environmental, social, and governance (ESG) issues as well, leading to an increased purchase intention toward products with the pro-environmental prompts. Before delving deeper into this study, we will go over some key aspects of the existing literature on the concept of the environmental prompts and COVID-19 concerns and their potential effects on consumer behavior.

## Literature Review and Hypotheses

### Environmental Prompt

Two approaches were adopted in previous literature to account for pro-environmental behavior: personal and situational. The personal approach attempts to identify individual characteristics, including environmental attitudes, demographic factors, and personality constructs. The situational approach attempts to identify environmental aspects that enhance pro-environmental behaviors, including prompting, rewards, and commitment (Schultz et al., 1995). Prior study has investigated personal and situational variables separately as well as interactions between these variables.

Extant study focusing on the situational approach has suggested that prompts or reminders of when to execute the desired action is an important situational factor that effectively fosters pro-environmental behaviors (Moussaoui et al., 2020). For example, a meta-analysis of 44 articles on prompts has reported that the moderate-to-high effect of environmental prompts enhances pro-environmental actions, including public energy conservation and public recycling (Osbaldiston and Schott, 2012). In addition, environmental prompts increase community stair usage across various commuter settings (Dolan et al., 2006). Likewise, prompts perform best for simple and repeated actions, particularly when prompts are presented explicitly in the context where the user should act (Schultz, 2014). This manner of prompt is described as a point-of-decision prompt in that it appears at the precise moment and location when the user must decide whether or not to conduct the behavior. Prompts are useful when people simply forget to behave or are distracted by other stimuli. In these cases, prompts can serve as reminders of pro-environmental behavior. Therefore, it would be useful to investigate the effects of prompts in a certain situational context such as overwhelming crises where people are distracted and easily forget to do good deeds for others.

In this study, we have focused on the ongoing COVID-19 pandemic and investigated the interaction between the individual pandemic concerns and the presence of prompts to evoke pro-environmental behaviors. To the best of our knowledge, few empirical studies have directly examined the link between COVID-19 pandemic and prompts on pro-environmental behaviors. Although a large body of extant literature has tested the effects of prompts on individual environmental attitudes or behavioral changes (Wiese et al., 2004; Kurz et al., 2005), relatively little study has investigated them in the context of COVID-19 pandemic. Since COVID-19 outbreak has altered individual and collective behavior changes (Ramkissoon, 2020), it is worth noting that this study provides a deeper understanding of ways to promote pro-environmental actions in the midst of COVID-19 pandemic.

### Coronavirus Disease 2019 Concerns (Altruistic vs. Egoistic)

Worry is described as a bothersome mind where the state of an issue in a certain domain of life departs from its desired state (Boehnke et al., 1998). Worriers continue to worry despite the fact that it is a nasty experience characterized by a flow of negative images and thoughts, unpleasant emotions, and a loss of mental control (Mathews, 1990). Worry includes daily concerns as well as severe and extended problems that may be linked to an anxiety disorder diagnosis (Borkovec et al., 2004). Previous literature has suggested the concept of intolerance uncertainty in relation to concern (Freeston et al., 1994; Boehnke et al., 1998). Individuals who worry strive to exercise some control over the circumstances they confront in life in the hope of preventing or diminishing future negative consequences. Therefore, worry and the ability to tolerate uncertainty are negatively correlated (Ladouceur et al., 2000).

Coronavirus disease 2019 crisis can cause people to feel stressed and worried, not only as a threat to survival, but also because of the restrictions in social activities and disruption to social networks, such as social distancing, quarantine, and work closures (Limcaoco et al., 2020). Worry is known to be one of the most common responses to the outbreak of COVID-19 crisis and recent articles have investigated the antecedents or consequences of individuals’ concerns in this difficult time (Zysberg and Zisberg, 2020). For example, recent studies have demonstrated that higher social status, larger family size, a greater sense of community, and little knowledge of the pandemic have lowered concerns as buffers during COVID-19 pandemic (Meltzer et al., 2021; Rojas-Méndez, 2021). Zhou and Guo (2021) has revealed the outcome of different types of concerns evoked by COVID-19 pandemic by demonstrating that economic concerns and safety concerns do not predict the death rate during COVID-19 pandemic, whereas health concerns result in a decrease in fatality in COVID-19 crisis.

As an unmanageable crisis that poses a threat to human safety and existence, we assume that concerns related to COVID-19 may influence pro-environmental attitude and behavior. Environmental protection has been described by some scholars as a “luxury good” that is appealing when the situation is normal and well-off but being ignored during times of difficulties (Abou-Chadi and Kayser, 2017). For example, economic crises such as the Great Recession of 2008 have a detrimental impact on individuals’ willingness to pay for climate change prevention (Ivlevs, 2019). Similarly, unemployment rates have negative impacts on individuals prioritizing environmental preservation (Kenny, 2020). Therefore, we expect that evoked concerns during the difficult times would lead to decreased attention to others and environment.

**Hypothesis 1:** People will show less purchase intention toward products with environmental prompt (e-prompt) when recalling COVID-19 concerns compared to the normal situation.

Furthermore, we considered the altruistic and egoistic values in determining individuals’ concerns evoked by the pandemic (Yadav, 2016). Extant literature has demonstrated that altruistic values (concern for others) and egoistic values (concern for the self) are the two fundamental motives to behave in a virtuous way (Schwartz, 1992). The term “altruistic” refers to a condition in which individuals behave on behalf of others without any personal gain (Schwartz, 1977). On the other hand, the term “egoistic” refers to acting on one’s own behalf or alleviating one’s own pain and damage (Stern et al., 1993). Based on past literature, we have termed altruistic COVID-19 concerns as concern for others, such as concerns about impairment of the national economy or national health, whereas we have described egoistic COVID-19 concerns as concern for self during the pandemic, including concerns about the disadvantages of individual health or financial loss (Prakash et al., 2019; Rojas-Méndez, 2021).

We expect that the types of COVID-19 concerns would influence pro-environmental behaviors differently. Altruistic values or concerns are critical in molding consumer behavior toward the environment (Heberlein, 1972). Individuals with altruistic concerns behave for the welfare of others without seeking personal benefit (Yadav, 2016). On the other hand, egoistic values or concerns motivate individuals to perform in their own interest (De Groot et al., 2013). Individuals with self-centered considerations swiftly displace their behavior depending on the gains and expenses (Diekmann and Preisendörfer, 2003). Therefore, egoistic values do not lead to pro-environmental behavior without guaranteed advantages. As stated earlier, extant literature has demonstrated the inconsistent findings whether COVID-19 enhances or reduces prosocial behaviors including the environmental domain. We assume that these mixed findings may result from the different types of COVID-19 concerns. Previous literature has measured COVID-19 concerns without distinction of content [Amato et al., 2021; “Currently, how concerned are you about coronavirus/COVID-19?”]. By suggesting the different impacts of types of COVID-19 concerns on consumer behavior, this study tried to reconcile the previous inconsistent findings. We expect COVID-19 concerns for others and communities to lead to an increased pro-environmental behavior, whereas COVID-19 concerns for the self would not change consumer behavior toward the environment. Therefore, we hypothesize the following:

**Hypothesis 2:** People will show greater purchase intention toward the e-prompt products (vs. without the e-prompt products) when recalling altruistic COVID-19 concerns. The presence/absence of e-prompts does not affect purchase intention when recalling egoistic COVID-19 concerns.

### Environmental, Social, and Corporate Governance

The growing number of firms implementing sustainability plans and disclosing ESG data has promoted fundamental shifts in business models and management theory (Xie et al., 2019). The aim of traditional shareholder-oriented management is to maximize shareholder advantages and improve financial performance (Friedman, 1970). Sustainable management, on the other hand, focuses on minimizing externalities and optimizing social values regarding ESG issues considering all the shareholders, communities, consumers, and other related organizations. Recent reports have demonstrated the role of ESG performance and confirmed its accumulative importance during COVID-19 pandemic (Broadstock et al., 2021). Emerging evidence supports the notion that sustainability firms have fewer downside risks and are more robust during times of crisis (Hoepner et al., 2019; Jacobsen et al., 2019). Therefore, it is fundamental to investigate ways to increase ESG concerns among consumers during COVID-19 period.

**FIGURE 1:**
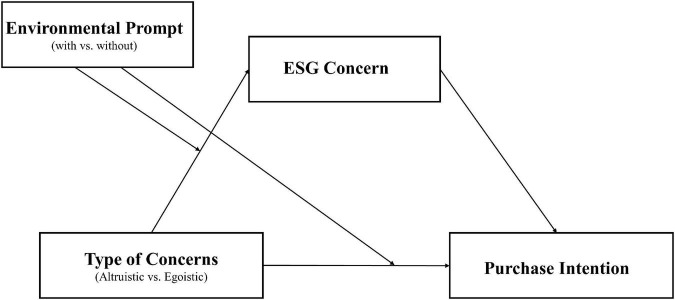
Conceptual framework.

Prior literature has demonstrated that altruistic motives and values are essential in shaping individual behaviors toward the environment and the welfare of communities (Prakash et al., 2019). Specifically, Romani et al. (2013) showed that individuals with altruistic values behave more favorably toward companies practicing sustainable values. Similarly, we expected that consumers with concerns about the national economy or health during the pandemic, considered an altruistic value, will lead to greater ESG concerns. Finally, an increased ESG concerns will lead to greater purchase intention toward the e-prompt products, as consumers with greater awareness of corporate social responsibility (CSR) are more likely to purchase socially responsible products (Pomering and Dolnicar, 2009; Tian et al., 2011). In addition, Kang et al.’s (2012) study directly supports our assumption that people with greater degrees of environmental concern indicate an increased willingness to pay premiums for hotels’ green initiatives. This positive link between environmental concern and willingness to pay for green products has been found in other contexts, such as ecolabeled appliances and furniture (2012) and environment-friendly food products (Shin et al., 2019). Thus, it is plausible that ESG concern, an extended concept of environmental concern, will have a positive effect on consumers’ green consumption. On the other hand, egoistic COVID-19 concerns will not influence individuals’ ESG concerns and purchase intention toward environmental products because self-centered values lead individuals to behave in their own interest (Yadav, 2016). Yeh et al. (2014) reported that egoism is one of the significant obstacles to promote CSR implementation. Therefore, this study proposes the following hypotheses and visualizes the study model (see Figure 1):

**Hypothesis 3:** Concerns about environmental, social, and corporate governance (ESG) will mediate the interaction effect between the type of COVID-19 concerns and the presence/absence of e-prompt products.

## Study 1: COVID-19 Concerns and Pro-Environmental Products

Both the study 1 and study 2 were conducted in accordance with the ethical standards of the Institutional Review Board of Seoul Women’s University, Seoul, South Korea (IRB 2021A-43). Study 1 was designed to determine the effects of COVID-19 on pro-environmental product consumption. Consistent with H1, we predict that people would indicate lower purchase intention toward pro-environmental products than normal products when recalling COVID-19 concerns.

### Materials and Methods

#### Participants, Design, and Procedure

A total of 117 participants from the US (77 women; *M*_age_ = 30.97, *SD* = 10.57) were recruited *via* the Prolific Academic online panel service,^1^ see detailed demographic information in Table 1. We chose the sample size based on the G*Power program (Faul et al., 2007). For this study, based on the input parameters (effect size *f* = 0.40, α error probability = 0.05, power = 0.80, and number of groups = 2), choosing a total sample size of 52 was recommended (Faul et al., 2007). The participants were each randomly assigned to one of the two conditions as either recalling or not recalling COVID-19 concerns. In the condition of recalling COVID-19 concerns, participants were guided in the instruction to write down sentences including the phrases (e.g., concerns for the country’s economy, the safety of my family) (Rojas-Méndez, 2021). For the normal condition, participants were guided to write what they did for today. There was no time or length limit, but they were instructed to write down at least five sentences in both the conditions. After completing the writing task, participants responded to two 7-point scales to provide their purchase intention toward a notebook with e-prompt (Yan et al., 2021): “How inclined would you be to purchase this notebook?” and “How willing would you be to purchase this notebook?” (1 = not at all and 7 = very much). The e-prompt for the notebook was presented with the label of “recycled.” Thus, a notebook with the label “recycled” is considered to be a product with an e-prompt and a notebook without the label “recycled” is considered to be a product without an e-prompt. Further, both the types of notebooks were presented at the same price of $ 3 by following the study of Yan et al. (2021). Scores on these two items of purchase intention were averaged to form a composite purchase intention scale (α = 0.92). Then, participants responded to two 7-point scales of manipulation check questions to find out they perceived the writing task as we intended: “The previous writing task was related to the concerns of COVID-19” and “The previous writing task *was not* related to the concerns of COVID-19” (1 = not at all and 7 = very much). Finally, the questions to collect demographic information were asked and a debriefing session was followed after the survey was completed.

**TABLE 1:** Sample demographic for study 1.

	Study 1	
Variables	Frequency (*N*)	Percent (%)
**Gender**		
Male	40	34.19
Female	77	65.81
**Age**		
≤20s	60	51.28
30s	35	29.91
≥40s	22	18.80
**Education degree**		
Less than high school	1	0.86
High school	9	7.69
Some college	34	29.06
2-years college	12	10.26
4-years college	39	33.33
Master’s degree	16	13.68
Professional degree	3	2.56
Doctoral degree	3	2.56
**Yearly income (unit: 000 USD)**		
≤29	55	47.01
30–49	17	14.53
50–89	28	23.93
≥90 and above	17	14.53
**Employment status**		
Employed full-time	48	41.03
Employed part-time	15	12.82
Unemployed/looking for work	25	21.37
Student	18	15.38
Homemaker	10	8.55
Retired	1	0.85
Total	117	100

### Results

#### Manipulation Checks

A one-way ANOVA on the manipulation check for recalling/not recalling COVID-19 concerns indicated that participants who were involved in the writing task to include phrases of COVID-19 concerns marked higher scores on the first manipulation check question than those who were guided to write down what they did for today [*M*_COVID–19 condition_ = 6.54 vs. *M*_normal condition_ = 1.54; *F*_(1,115)_ = 628.85, *p* < 0.001]. The same ANOVA for the second manipulation check question showed the reversed results compared to the first manipulation check question. Participants who were involved in the writing task instructed to include phrases related to COVID-19 concerns marked lower scores on the second manipulation check question than those who were guided to write down their daily life of that day [*M*_COVID–19 condition_ = 1.60 vs. *M*_normal condition_ = 6.46; *F*_(1,115)_ = 437.51, *p* < .001]. Thus, we can confirm that participants perceived the writing task in the way we intended.

#### Purchase Intention

**FIGURE 2:**
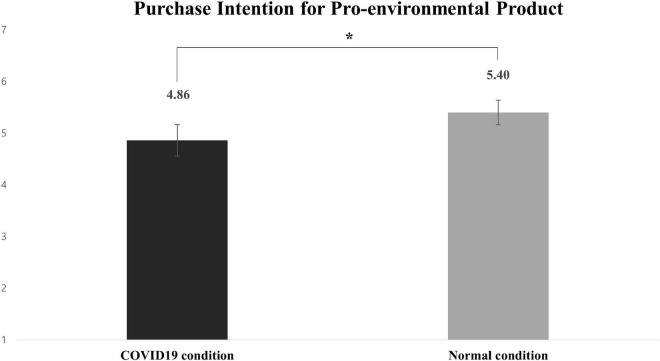
Effects of recalling coronavirus disease 2019 (COVID-19) on purchase intention for pro-environmental product (study 1). Error bars show the 95% CIs around the means. *p < 0.1.

A one-way ANOVA on purchase intention revealed that the main effect of recalling/not recalling COVID-19 concerns was significant [*M*_COVID–19 condition_ = 4.86 vs. *M*_normal condition_ = 5.40; *F*_(1,115)_ = 3.62, *p* = 0.06] (see Figure 2).

### Discussion

As we predicted, participants show less purchase intention toward pro-environmental products when recalling COVID-19 concerns than the condition of not recalling COVID-19 concerns. However, while reviewing the experiences, we found that the concerns for COVID-19 were mixed with altruistic and egoistic COVID-19 concerns—the portions of egoistic COVID-19 concerns were much higher than altruistic ones (78%, 49 out of 63 participants). For example, the examples of egoistic concerns are as follows: “I am worried that I will not be able to get a job with good enough pay to support myself,” “When the outbreak first happened, there was a toilet paper shortage and I was worried about not getting the supplies I needed,” and “I concern about not being able to make ends meet because rent prices are drastically increasing and my income is not.” On the other hand, altruistic concerns focus more on concerns for others: “I have also been worried that I could contribute to spreading the virus to others in my community who are vulnerable,” “I am concerned about the country’s economy because the ongoing pandemic is holding back businesses,” and “Antivaxxers are contributing to the spread of the virus and jeopardizing overall public health.” Thus, in study 2, we disentangled COVID-19 concerns from altruistic to egoistic and manipulated two different types of COVID-19 concerns in order to take a closer look at the consumer behavior on the basis of each type of concern.

## Study 2: Altruistic and Egoistic Concerns of COVID-19 and Pro-Environmental Products

Study 2 was designed to determine the effects of two types of COVID-19 concerns on pro-environmental product consumption. We predicted that people would show different attitudes toward pro-environmental products depending on the different types of COVID-19 concerns. We also predicted that people would show greater purchase intention toward the e-prompt products (vs. without the e-prompt products) when recalling altruistic COVID-19 concerns and the presence or absence of e-prompts would not affect purchase intention when recalling egoistic COVID-19 concerns, consistent with H2. Further, study 2 was designed to determine the underlying mechanism to explain the effects observed in H2. We predicted that concerns for ESG would mediate the interaction effect between the type of COVID-19 concerns and with/without the e-prompt products.

### Materials and Methods

#### Participants, Design, and Procedure

A total of 100 participants from the US (73 women; *M*_age_ = 30.83, *SD* = 11.48) were recruited *via* the Prolific Academic online panel service, see detailed demographic information in Table 2. Based on the input parameters (effect size *f* = 0.40, α error probability = 0.05, power = 0.80, and number of groups = 4), a total sample size of 76 was recommended (Faul et al., 2007). Participants were randomly assigned to one of four conditions in a 2 (types of COVID-19 concerns: altruistic vs. egoistic) × 2 (products: with e-prompt vs. without e-prompt). In the condition of recalling COVID-19 concerns related to the altruistic issues, participants were guided to write down sentences including the phrases (e.g., worry for the world’s economy, overall public health, and the safety of the people in the world) in the instruction (Rojas-Méndez, 2021). For COVID-19 concerns related to the egoistic issues, participants were guided to write down sentences including the phrases (e.g., my job or income, my personal health, and safety of myself or my family) in the instruction (Rojas-Méndez, 2021). There was no time or length limit, but they were instructed to write down at least five sentences in both the conditions. After completing the writing task, participants responded to three 7-point scales to provide their attitude toward the products with/without the e-prompt products (1 = very negative, 7 = very positive; 1 = very bad, 7 = very good; and 1 = unfavorable, 7 = favorable) (Batra and Ahtola, 1991; Folkes and Kamins, 1999). Scores on these three items were averaged to form a composite attitude scale (α = 0.90). The e-prompt for the notebook was presented with the label of a “recycled” exactly the same as in study 1. Further, participants responded to the same two 7-point scales utilized in study 1 to provide their purchase intention (Yan et al., 2021). Scores on these two items were averaged to form a composite purchase intention scale (α = 0.92). In addition, the participants answered a 7-point scale to provide their perceived concerns for ESG: “During COVID-19 pandemic, I had more chances to think about those topics: environmental, social, and corporate governance and sustainability” (1 = not at all and 7 = very much) [adapted from Kang et al. (2012)]. Then, participants responded to two 7-point scales of manipulation check questions to find out they perceived the writing task as we intended: “The previous writing task was related to the *self-concerns* of COVID-19” and “The previous writing task was related to the *social concerns* of COVID-19” (1 = not at all and 7 = very much). The manipulation checks for the product with/without e-prompt were followed. The participants responded to three 7-point scales to provide their perception regarding the notebook presented in the experiment: “The notebook is an environmental-friendly product,” “The notebook is a green product,” and “The notebook is beneficial to the environment” (1 = not at all and 7 = very much). Scores on these three items were averaged to form a composite manipulation check scale for the product with/without e-prompt (α = 0.88). Finally, the questions to collect demographic information were asked and a debriefing session was followed after the survey was completed.

**TABLE 2:** Sample demographic for study 2.

	Study 2	
Variables	Frequency (*N*)	Percent (%)
**Gender**		
Male	27	27.00
Female	73	73.00
**Age**		
≤20s	58	58.00
30s	26	26.00
≥40s	16	16.00
**Education degree**		
High school	6	6.00
Some college	26	26.00
2-years college	7	7.00
4-years college	46	46.00
Master’s degree	10	10.0
Professional degree	5	5.00
**Yearly income (unit: 000 USD)**		
≤29	41	41.00
30–49	20	20.00
50–89	29	29.00
≥90 and above	10	10.00
**Employment status**		
Employed full-time	51	51.00
Employed part-time	13	13.00
Unemployed/looking for work	11	11.00
Student	17	17.00
Homemaker	5	5.00
Retired	3	3.00
Total	100	100

### Results

#### Manipulation Checks

A 2 × 2 ANOVA on the manipulation check for recalling COVID-19 concerns indicated that participants who were involved in the writing task of *self-concerns* of COVID-19 marked higher scores on the first manipulation check question than those who were guided to write down sentences including phrases of *social concerns* of COVID-19 [*M*_altruistic concerns_ = 1.80 vs. *M*_egoistic concerns_ = 6.47; *F*_(1,96)_ = 295.724, *p* < 0.001]. The same ANOVA for the second manipulation check question showed the reversed results compared to the first manipulation check question. Participants who were involved in the writing task of *social concerns* of COVID-19 marked higher scores on the second manipulation check question than those who were guided to write down *self-concerns* of COVID-19 [*M*_altruistic concerns_ = 6.45 vs. *M*_egoistic concerns_ = 3.24; *F*_(1,96)_ = 72.056, *p* < 0.001]. Thus, we can confirm that participants perceived the writing task in the way we intended. In addition, a 2 × 2 ANOVA on the manipulation check for the product with/without e-prompt showed that participants who were involved in the condition with e-prompt showed higher scores on the manipulation check scale for the product without e-prompt [*M*_with e–prompt_ = 5.88 vs. *M*_without e–prompt_ = 3.41; *F*_(1,96)_ = 83.998, *p* < 0.001].

#### Consumer Attitude

A 2 × 2 ANOVA on the consumer attitude indicated that the main effects of the product with/without e-prompt was significant [*M*_with e–prompt_ = 5.54 vs. *M*_without e–prompt_ = 4.97; *F*_(1,96)_ = 5.296, *p* = 0.024] and types of COVID-19 concerns were not significant [*M*_altruistic concerns_ = 5.15 vs. *M*_egoistic concerns_ = 5.40; *F*_(1,96)_ = 1.092, *p* = 0.299]. More importantly, the two-way interaction was significant [*F*_(1,96)_ = 3.753, *p* = 0.056]. Planned contrast indicated that consumer attitude toward the e-prompt products was significantly higher than without the e-prompt products when participants recalled social concerns of COVID-19 [*M*_with e–prompt_ = 5.65 vs. *M*_without e–prompt_ = 4.63; *t*_(96)_ = 2.981, *p* = 0.004]. However, consumer attitude did not vary depending on self-concerns of COVID-19 [*M*_with e–prompt_ = 5.44 vs. *M*_without e–prompt_ = 5.35; *t*_(96)_ = 0.259, *p* = 0.796].

#### Purchase Intention

**FIGURE 3:**
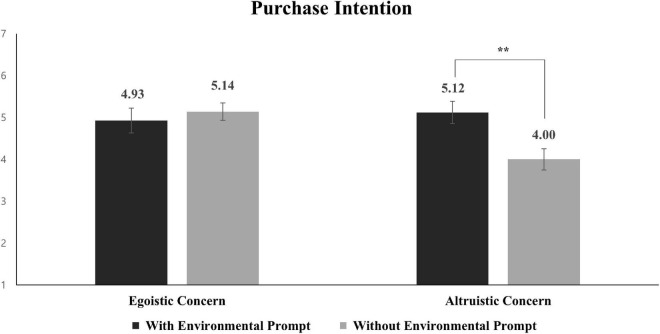
Effects of type of concerns and with/without environmental prompt product on purchase intention (study 2). Error bars show the 95% CIs around the means. **p < 0.05.

A 2 × 2 ANOVA on the purchase intention revealed that the both main effects of the product with/without e-prompt and types of COVID-19 concerns were not significant [*M*_with e–prompt_ = 5.02 vs. *M*_without e–prompt_ = 4.54; *F*_(1,96)_ = 1.875, *p* = 0.174; *M*_altruistic concerns_ = 4.57 vs. *M*_egoistic concerns_ = 5.02; *F*_(1,96)_ = 2.011, *p* = 0.159]. More critically, the two-way interaction was significant [*F*_(1,96)_ = 3.936, *p* = 0.050]. Planned contrast indicated that purchase intention toward the e-prompt products was significantly higher than without the e-prompt products when participants recalled social concerns of COVID-19 [*M*_with e–prompt_ = 5.12 vs. *M*_without e–prompt_ = 4.00; *t*_(96)_ = 2.358, *p* = 0.020]. However, purchase intention toward the e-prompt products did not vary depending on when participants recalled self-concerns of COVID-19 (*M*_with e–prompt_ = 4.93 vs. *M*_without e–prompt_ = 5.14; *t*_(96)_ = –0.437, *p* = 0.663) (see Figure 3).

#### Mediation Analysis

To test whether perceived concerns for ESG mediate the interaction effect between the type of COVID-19 concerns and the presence/absence of e-prompt products, we employed a bootstrapping analysis using the PROCESS version 3.0 macro (model 8) with 5,000 resamples (Hayes, 2017). The model uses the types of COVID-19 concerns as the independent variable (1 = egoistic concern and 0 = altruistic concern), the presence/absence of e-prompt products as moderator (1 = with the e-prompt products and 0 = without the e-prompt products), concerns for ESG as the proposed mediator, and purchase intention as the dependent variable. The overall mediation effect of concerns for ESG was found to be significant [90% CI = (–0.8412, –0.0305)]. In addition, the conditional indirect effect of the types of COVID-19 concerns on purchase intention was only significant with the e-prompt products condition [90% CI = (–0.6113, –0.1022)], but not without the e-prompt products condition [90% CI = (–0.2075, 0.3335)].

### Discussion

The results of study 1 were replicated in study 2. Consistent with H2, we confirmed that participants evaluated products with e-prompts more positively (vs. without the e-prompt products) when recalling altruistic COVID-19 concerns. The presence/absence of e-prompts did not affect purchase intention when recalling egoistic COVID-19 concerns. Furthermore, supporting H3, we corroborated that concerns for ESG mediated the moderating effect of with/without the e-prompt products on the asymmetrical pattern in purchase intention between the two types of COVID-19 concerns.

## General Discussion

This study explored the effects of COVID-19 on pro-environmental product consumption. Two experimental studies manipulated individuals’ COVID-19 concerns and the presence/absence of pro-environmental prompts. We found that consumers indicated lower purchase intention for the pro-environmental products when recalling COVID-19 concerns compared to normal situations (study 1). Further, by disentangling egoistic COVID-19 concerns (e.g., concerns about disadvantages to individuals’ work and finances) from altruistic COVID-19 concerns (e.g., concerns about damage to the country’s economy), we investigated the effects of both COVID-19 concerns on pro-environmental product consumption. We found that consumers reported an increased purchase intention toward products with the e-prompt products when manipulating altruistic COVID-19 concerns. However, the presence/absence of the e-prompt products did not affect consumers’ purchase intentions when recalling egoistic COVID-19 concerns (study 2). The concern for ESG issues mediated the interaction effect between the type of COVID-19 concerns and the presence/absence of the e-prompt products.

Although prosocial behaviors are fundamental for a sustainable society, little study has been conducted to directly determine which factors of COVID-19 accelerate or inhibit prosocial behaviors. Thus, in this study, we conducted pioneering study on pro-environmental behavior as a part of prosocial behavior by investigating when and why consumers show an increased or decreased pro-environmental behavior by applying consumers’ psychological factors, which appear to be most vulnerable to social crises such as COVID-19 pandemic era. What we learned from this study is that consumers’ concerns regarding COVID-19 include not only egoistic concerns, but also altruistic concerns and manipulating the specific type of COVID-19 concerns is possible. Finally, among the concerns, only altruistic COVID-19 concerns significantly amplified consumers’ pro-environmental behavior and the underlying mechanism to explain this phenomenon was found to increase ESG concerns. People who were manipulated with altruistic concerns (vs. egoistic concerns) showed an increased concern for ESG issues, which finally accounted for an increased purchase intention for pro-environmental products. Thus, characteristics of COVID-19 concerns should be focused on, depending on which sector the marketers belong to. Further, the first proposed ESG concern is meaningful as a novel concept, but the authors admit that it has not been defined clearly. Thus, we expect further study to expand this study.

Specifically, the study results provide several theoretical contributions. First, we empirically found that people have different types of concerns regarding COVID-19 pandemic (Limcaoco et al., 2020): not only egoistic concerns focusing on maximizing one’s outcomes, but also altruistic concerns reflecting issues for the welfare of others. Depending on the type of concerns they are manipulated with, consumers show an increased or decreased pro-environmental product consumption. Thus, this finding theoretically expends prosocial behavior literature that suggests that the nature of COVID-19 concern is a critical factor in accelerating or inhibiting consumers’ prosocial behavior. Second, the results demonstrate the concern for ESG issues as a psychological mechanism to understand why the two types of COVID-19 concern affect prosocial behavior differently. They also show that people who are manipulated with altruistic concerns (vs. egoistic concerns) show an increased concerns for ESG issues, which finally account for an increased purchase intention for pro-environmental products.

The results of this study suggest practical implications for marketers in the field. Among the types of concerns regarding COVID-19 pandemic, this study proves that manipulating specific types of concerns is possible. In addition, different effects on pro-environmental behavior were observed depending on the type of COVID-19 concerns. Thus, during the process of communicating with consumers, marketers can emphasize marketing messages that can take advantage of persuading consumers more effectively. For example, in the field of prosocial marketing contexts or public institutions, marketers can highlight the altruistic issues related to COVID-19 pandemic, which may help to increase consumers’ prosocial intentions. As we observed in this study, being manipulated with altruistic concerns (vs. egoistic concerns), evokes increased concern for ESG issues, which finally account for an increased purchase intention for pro-environmental products. On the other hand, it would be useful for marketers, belonging to private companies, to emphasize consumers’ egoistic concerns focusing on maximizing one’s outcomes when persuading consumers in the general consumer goods sector. In addition, even in COVID-19 catastrophe, we found that one of the situational factors of the environmental prompt works as an effective way to communicate pro-environmental behaviors to consumers. In other words, consumers can differentiate pro-environmental products from the existence of e-prompts. Thus, marketers in the field could consistently utilize e-prompts when delivering the message to consumers that they provide products or services that are environment-friendly, even in COVID-19 pandemic situation.

Despite its substantial theoretical and field contributions, this study has several limitations. First, it used a single item as an exploratory attempt to measure ESG concerns. The ESG concern item directly assesses individuals’ concerns about ESG issues based on the definition (Corporate Finance Institute, 2022). However, future study based on a more comprehensive measurement to assess ESG concerns would provide us with more defined and definitive conclusions. Second, this study classified COVID-19 concerns into two types: altruistic and egoistic. Although they are two principle drives to behave in the desired way (Schwartz, 1992), future study can use more detailed classifications of COVID-19 concerns, including economic, safety, and health concerns, as Rojas-Méndez (2021) has suggested. Third, we employed the 2 × 2 between-subject design and, therefore, we cannot draw conclusions about causal relationships between variables including egoistic COVID-19 concerns that lead to altruistic COVID-19 concerns and vice versa. To address this issue, longitudinal studies using a variety of designs, such as the experience sampling method or the daily diary method, are needed in future study. Fourth, regarding the design of the studies, we tested two conditions of COVID-19 concern (vs. no-concern) in study 1, while in study 2, we only tested two types of concerns, altruistic vs. egoistic, rather than including no-concern condition. We understand including the no-concern condition in study 2 would have been helpful to extend the idea of study 1. Thus, to give better insights for readers with a holistic point of view, we will include the no-concern condition in the design of the experiment in further studies. Last, to capture individuals’ pro-environmental behaviors, we measured the attitudes and purchase intentions of the e-prompt product. Future study can broaden the applicability of the findings by investigating environment-friendly behaviors in a variety of domains, such as measuring intention to participate in the pro-environmental campaign or willingness to invest time and money in pro-environmental behavior.

## Data Availability Statement

The datasets generated for this study are available on request to the corresponding author.

## Ethics Statement

The studies involving human participants were reviewed and approved by Seoul Women’s University. The participants provided their written informed consent to participate in this study.

## Author Contributions

Both authors jointly designed the experiments, undertook data collection, analyzed, and wrote up this manuscript.

## Conflict of Interest

The authors declare that the research was conducted in the absence of any commercial or financial relationships that could be construed as a potential conflict of interest.

## Publisher’s Note

All claims expressed in this article are solely those of the authors and do not necessarily represent those of their affiliated organizations, or those of the publisher, the editors and the reviewers. Any product that may be evaluated in this article, or claim that may be made by its manufacturer, is not guaranteed or endorsed by the publisher.

## Footnotes

1
www.prolific.ac
