# Supplementary material for: How Can We Increase Pro-environmental Behavior During COVID-19 Pandemic? Focusing on the Altruistic (vs. Egoistic) Concerns
Source: Front Psychol. 2022 May 3;13:870630. doi: 10.3389/fpsyg.2022.870630 (PMC9110922; doi:10.3389/fpsyg.2022.870630)
Supplement: Supplementary file 1 [file Data_Sheet_1.docx]

**Appendix**

**A. Study Design and Stimuli**

| Study Detail | Explanation | |
| --- | --- | --- |
| Study 1 | | |
| Design | 2 conditions: (COVID 19 condition vs. Normal condition)  Dependent variable: Purchase intention toward products | |
| Scenario | COVID 19 condition:  Please follow the instructions. Following phrases are the most popular concerns related to the covid-19.  Please write down sentences with these phrases.  There is no time restriction or specific format. Please take your time and write down at least five sentences.  Economic Worries  - Worry for the Country’s Economy  - Not Being Able to Get the Supplies I Need  - Negative Impact on My Job or Income  -  Not Being Able to Make Ends Meet  Health Worries  - Overall Public Health  - Health of My Relatives in Vulnerable Populations  - My Personal Health  - Contributing to Spread the Virus  Safety Worries  - Safety of Myself or My Family  - Taking Care of My Family | |
|  | Normal condition:  Please follow the instructions. Please write down what you did for today.  There is no time restriction or specific format.  Please take your time and write down at least five sentences. | |
| Product stimuli | 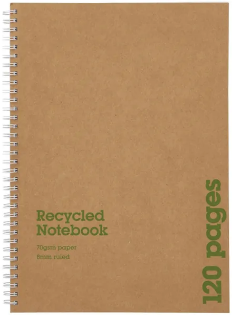With e-prompt products:  Recycled Notebook ($3) |  |
| Study 2 | | |
| Design | 4 conditions: 2 (types of COVID-19 concerns: altruistic vs. egoistic) × 2 (products: with e-prompt vs. without e-prompt)  Dependent variable: Purchase intention toward products  Mediating variable: Perceived concerns for ESG | |
| Scenario | COVID 19 egoistic concern:  Following phrases are the most popular personal concerns related to the covid-19. Think about the covid-19 as a personal concern.  Please write down sentences with these phrases.  Personal Economic Worries - Negative Impact on My Job or Income  Personal Health Worries - My Personal Health   Personal Safety Worries - Safety of Myself or My Family  There is no time restriction or specific format. Please take your time and write down at least five sentences. | COVID 19 altruistic concern:  Following phrases are the most popular social concerns related to the covid-19. Think about the covid-19 as a social concern, not just a personnel concern.    Please write down sentences with these phrases.    Social Economic Worries - Worry for the World’s Economy  Social Health Worries - Overall Public Health   Social Safety Worries - Safety of the people in the world  There is no time restriction or specific format. Please take your time and write down at least five sentences. |
| Product stimuli | With e-prompt products:  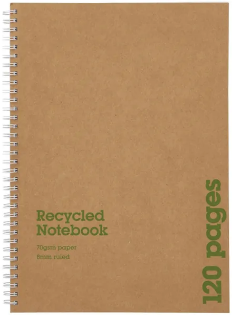Recycled Notebook ($3) | Without e-prompt products:  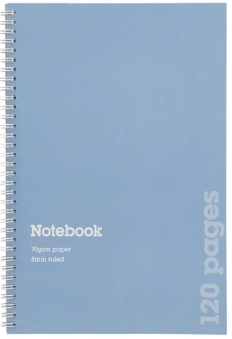Regular Notebook ($3) |

**B. Constructs and Items**

| Construct | Items |
| --- | --- |
| Study 1 | |
| Purchase intention | How inclined would you be to purchase this notebook?  How willing would you be to purchase this notebook?  (1 = not at all, 7 = very much) |
| Manipulation check for COVID-19 concerns | The previous writing task was related to the concerns of COVID-19.  The previous writing task *was not* related to the concerns of COVID-19.  (1 = not at all, 7 = very much) |
| Study 2 | |
| Attitude toward the products | 1 = very negative, 7 = very positive  1 = very bad, 7 = very good  1 = unfavorable, 7 = favorable |
| Purchase intention | How inclined would you be to purchase this notebook?  How willing would you be to purchase this notebook?  (1 = not at all, 7 = very much) |
| Perceived concerns for ESG | During the COVID-19 pandemic, I had more chances to think about those topics; environmental, social, and corporate governance and sustainability.  (1 = not at all, 7 = very much) |
| Manipulation check for types of COVID-19 concerns | The previous writing task was related to the *self-concerns* of COVID-19. The previous writing task was related to the *social-concerns* of COVID-19.  (1 = not at all, 7 = very much) |
| Manipulation check for the product with/without e-prompt | The notebook is an environmentally friendly product.  The notebook is a green product.  The notebook is beneficial to the environment.  (1 = not at all, 7 = very much) |
